# Long-term risk of subsequent cancer incidence among hereditary and nonhereditary retinoblastoma survivors

**DOI:** 10.1038/s41416-020-01248-y

**Published:** 2021-01-21

**Authors:** Sara J. Schonfeld, Ruth A. Kleinerman, David H. Abramson, Johanna M. Seddon, Margaret A. Tucker, Lindsay M. Morton

**Affiliations:** 1 Division of Cancer Epidemiology and Genetics, National Cancer Institute, Bethesda, MD USA; 2 Memorial Sloan Kettering Cancer Center, New York, NY USA; 3 University of Massachusetts Medical School, Worcester, MA USA

**Keywords:** Cancer epidemiology, Eye cancer, Paediatric cancer

## Abstract

**Background:**

Increased sarcoma and melanoma risks after hereditary retinoblastoma are well established, whereas less is known about epithelial subsequent malignant neoplasms (SMNs) and risks for multiple (≥2) SMNs.

**Methods:**

Leveraging long-term follow-up and detailed histologic information, we quantified incident SMN risk among 1128 hereditary and 924 nonhereditary retinoblastoma survivors (diagnosed 1914–2006; follow-up through 2016). Standardised incidence ratios (SIRs) compared cancer risk after retinoblastoma relative to the general population. We estimated cumulative incidence accounting for competing risk of death.

**Results:**

Hereditary survivors had statistically significantly increased SMN risk (*N* = 239; SIR = 11.9; 95% confidence interval [CI] 10.4–13.5), with SIRs >80-fold for sarcomas, nasal cavity tumours and pineoblastoma. Significantly increased risks were also observed for melanoma and central nervous system, oral cavity and breast SMNs (SIRs = 3.1–17), but not the uterus, kidney, lung, bladder, pancreas or other types. Cumulative incidence 50 years following hereditary retinoblastoma was 33.1% (95% CI 29.0–37.2) for a first SMN and 6.0% (95% CI 3.8–8.2) for a second SMN. SMN risk was not increased after nonhereditary retinoblastoma (*N* = 25; SIR = 0.8; 95% CI 0.5–1.2).

**Conclusion:**

Beyond the established sarcoma and melanoma risks after hereditary retinoblastoma, we demonstrate increased risk for a more limited number of epithelial malignancies than previously suggested. Cumulative incidence estimates emphasise long-term SMN burden after hereditary retinoblastoma.

## Background

Retinoblastoma is a rare retinal tumour that is primarily diagnosed during early childhood and is most commonly caused by mutations in the *RB1* tumour suppressor gene. Previous studies have demonstrated increased risks of subsequent malignant neoplasms (SMNs), which are particularly high for bone and soft tissue sarcomas and melanoma, and are largely restricted to hereditary survivors (i.e. patients carrying a germline mutation in the *RB1* gene).^1–6^ In addition to this genetic susceptibility, increased risks also are attributed to treatment, especially radiotherapy and subsequent sarcoma risks.^7^

The extent to which retinoblastoma survivors face increased risks for epithelial cancers is less clear. Previous studies have reported lower, but statistically significant increases for a number of epithelial cancers,^1,2,5,8^ but sample sizes have been small because of the long-term follow-up required to assess risk for these malignancies, which typically occur later in life.^9^ Incomplete histologic information (e.g. for cases ascertained from death certificates) and inclusion of sarcomas in site-specific groups also has made it difficult to distinguish epithelial tumours from sarcomas, particularly for sites where sarcomas are observed in this population, including nasal cavity and uterus (two common sites), as well as kidney and bladder.^2,10^ Thus, it remains unclear whether risks for epithelial tumours are indeed increased.

Several recent studies of survivors of Hodgkin lymphoma and other childhood cancers have highlighted the growing understanding that these patients face a lifelong risk for developing multiple SMNs.^11,12^ Third, fourth and even fifth SMNs have been reported in survivors of hereditary retinoblastoma,^13,14^ but the risks for developing multiple SMNs are not well quantified. Evaluation of risks for third- and higher-order primary malignancies is important for describing the burden of SMNs and may also help inform surveillance strategies in this population. To address these research gaps, we used data from the National Cancer Institute Long-Term Follow-up Study of Retinoblastoma Survivors to describe the long-term risks for a broad range of SMNs, leveraging detailed SMN diagnostic data with histologic information to evaluate risks for subsequent epithelial tumours and to quantify risk for multiple SMNs.

## Methods

### Study population

The study includes patients diagnosed with retinoblastoma between 1914 and 2006 at two major medical centres in New York and Boston.^5,10^ For patients diagnosed with retinoblastoma between 1914 to 1984, 1985 to 1996 and 1997 to 2006, medical records were reviewed in 1984, 1996 and 2006, respectively, to systematically capture patient characteristics, including diagnosis and treatment of retinoblastoma, family history of retinoblastoma at the time of retinoblastoma diagnosis and diagnosis of subsequent neoplasms. As germline *RB1* mutation status was not available for all patients, we classified patients as having hereditary retinoblastoma if they had bilateral retinoblastoma or unilateral disease and a family history of retinoblastoma. Individuals with unilateral retinoblastoma and no known family history at diagnosis were classified as having nonhereditary disease. After excluding individuals first seen >5 years after diagnosis of retinoblastoma (*N* = 29), those lacking follow-up (*N* = 10), and non-US residents (*N* = 45), the resulting study population included 2052 (1128 hereditary and 924 nonhereditary) survivors.

### Ascertainment and classification of SMNs

SMNs were ascertained during the initial medical record review, from periodic follow-up questionnaires between 1987 and 2016 (665/1128 [59%] of hereditary and 630/924 [68.2%] of nonhereditary survivors completed at least one questionnaire) and linkages with the National Death Index (NDI) through 2016. Self-reported SMNs from the questionnaires were then confirmed by pathology reports (and recurrences or metastases were excluded), and all SMNs were classified by type, separating sarcomas from epithelial malignancies (Supplementary Table S1). Cases missing histologic data (particularly common for mortality-based cases) were classified by organ using only topography code. We separated pineoblastoma from other central nervous system (CNS) tumours. Pineoblastomas typically develop within 5 years of retinoblastoma diagnosis^15^ and were first recognised as distinct from other CNS primaries and retinoblastoma metastases in the late 1970s.^16,17^ Because of the difficulties in distinguishing between recurrences and new primaries, subsequent diagnoses of retinoblastoma and cancers of the orbit (other than those classified as a sarcoma based on histologic information) were not counted as SMNs. We further excluded nonmelanoma skin cancers, which are not routinely collected by cancer registries and thus could not be included in comparisons with the general population.

The study was approved by the Special Studies Institutional Review Board at the National Cancer Institute. Survivors (or their legal guardians for survivors <18 years of age) provided informed consent for follow-up questionnaires.

### Statistical analysis

All analyses were stratified by hereditary status. For our primary analyses, patients were followed from the date of retinoblastoma diagnosis until the earliest of diagnosis date of the first incident cancer of interest for a given analysis (e.g. first breast cancer, irrespective of earlier SMNs) or censored at the date of last contact (defined as the most recent questionnaire or, for patients who never completed a questionnaire, the earliest of death or estimated medical record abstraction date).

Standardised incidence ratios (SIRs; ratio of the observed to expected cases) and confidence intervals compared SMN rates among retinoblastoma survivors with incident malignancy rates in the US general population (a measure of relative risk). Expected values were generated from SEER 9 (Surveillance, Epidemiology, and End Results Programme)^9^ (applying the classification used for observed cases) from 1975 to 2016 stratified by calendar year, sex and age and multiplied by stratum-specific person-years at risk in the cohort. Two-sided *P* values <0.05 (corresponding to exclusion of 1.0 from the confidence limit) were considered statistically significant. We also present the absolute excess risks per 10,000 person-years: [(observed − expected)/person-years] × 10,000. SIRs and AERs were calculated for all SMNs and specific types with at least three cases.

We also evaluated the absolute risk by calculating the cumulative incidence of SMNs (total and for specific types with at least ten cases), accounting for competing risk of death.^18^ A priori, we decided to estimate cumulative incidence for breast cancer among nonhereditary survivors regardless of sample size because previous analyses in this cohort suggested increased risk.^5,19^ In addition, we calculated the cumulative incidence of developing a third primary malignancy (total), following patients until the diagnosis of a third primary cancer (second SMN) or date of the last contact. Cumulative incidence of a third primary malignancy was estimated both within the full cohort (starting follow-up at the date of retinoblastoma) and restricted to individuals who developed a second primary (first SMN; starting follow-up at the date of the first SMN).

For all SMNs combined and types with at least ten cases, we evaluated the association between patient and treatment characteristics and SMNs using Cox proportional hazards models with time since retinoblastoma diagnosis (years) as the time scale. Because of the young age at retinoblastoma diagnosis, time since diagnosis and attained age are highly correlated, and thus these time scales yield virtually identical results. Statistical significance was based on two-sided Wald *P* values <0.05.

Because ascertainment of incident malignancies may be incomplete and also to facilitate comparison with previous studies, we included mortality-based cases in sensitivity analyses. Patients were followed until the first SMN of interest (based on the date of diagnosis or date of death for cancers ascertained from NDI/death certificates), death, or date of last known contact. Expected values for these SIR analyses included both incident and mortality-based cases in SEER.

In addition, because we lacked information on SMN treatments, which can influence the risk of later SMNs, we conducted sensitivity analyses censoring at diagnosis of any second primary cancer (first SMN), rather than continuing until the first SMN of interest. This changes the type-specific, but not total SMN, analyses. Diagnosis of other second primary cancer types (e.g. diagnosis of breast cancer in analyses of second primary melanoma) was a censoring event and treated as a competing risk in cumulative incidence analyses.

Statistical analyses were conducted using SAS v9.4 (Cary, NC).

## Results

Hereditary patients (*N* = 1128) were generally diagnosed with retinoblastoma at a younger age (median = 9.0 months) than nonhereditary patients (*N* = 924; median = 24.0 months; Table 1). Approximately 20% of hereditary survivors had a known family history of retinoblastoma. Whereas surgery was the primary treatment for most nonhereditary patients (68.8%), the majority of hereditary patients received radiation (primarily external beam radiotherapy), either alone (48.8%) or in combination with chemotherapy (38.6%). Median follow-up to first SMN or exit was 22.6 and 28.6 years, among hereditary and nonhereditary patients, respectively, with 10% of hereditary patients and 21% of nonhereditary patients exiting at attained ages ≥50 years.

**Table 1:** Selected characteristics of 1128 hereditary and 924 nonhereditary retinoblastoma survivors^a^.

	Nonhereditary		Hereditary	
	*N*	%	*N*	%
Total	924	100	1128	100
Age at retinoblastoma diagnosis (months)				
<12	187	20.2	642	56.9
12–23	266	28.8	310	27.5
24+	471	51.0	176	15.6
Median age at diagnosis	24.0 months		9.0 months	
Year of retinoblastoma diagnosis^b^				
<1960	212	22.9	304	27.0
1960–1969	224	24.2	297	26.3
1970–1979	204	22.1	248	22.0
1980–2006	284	30.7	279	24.7
Median year of diagnosis	1971		1968	
Sex				
Male	472	51.1	578	51.2
Female	452	48.9	550	48.8
Family history of retinoblastoma				
No/unknown	924	100.0	891	79.0
Yes	–	–	237	21.0
Treatment for retinoblastoma				
Radiation, no/unknown chemotherapy^c,d^	101	10.9	550	48.8
Radiation and chemotherapy^c^	86	9.3	435	38.6
Chemotherapy, no/unknown radiation^e^	61	6.6	39	3.5
No/unknown radiation/chemotherapy^f^	676	73.2	104	9.2
Surgery	636	68.8	90	8.0
Attained age (age at first SMN or exit)				
<20	299	32.4	497	44.1
20–<30	158	17.1	170	15.1
30–<40	139	15.0	199	17.6
40–<50	133	14.4	147	13.0
50+	195	21.1	115	10.2
Median person-years of follow-up^g^	28.6 years		22.6 years	
Developed SMN during follow-up^g^				
No	899	97.3	889	78.8
Yes	25	2.7	239	21.2

*SMN* subsequent malignant neoplasm, *EBRT* external beam radiotherapy.

^a^Hereditary status was determined based on medical records at the time of retinoblastoma diagnosis. Patients were classified as having hereditary retinoblastoma if they had bilateral retinoblastoma or unilateral disease and a family history of retinoblastoma. Individuals with unilateral retinoblastoma and no known family history at the time of retinoblastoma diagnosis were classified as having nonhereditary disease.

^b^Calendar year cut-points selected to yield roughly comparable group sizes as well as to correspond to the treatment era.

^c^Among the 187 nonhereditary survivors who received radiotherapy, 11 (5.9%) received brachytherapy only; 155 (82.9%) received external beam radiotherapy (EBRT) only; 8 (4.3%) received brachytherapy and EBRT; and 13 (7%) had unknown radiotherapy type. Among the 985 hereditary survivors who received radiotherapy, 37 (3.8%) received brachytherapy only; 815 (82.7%) received EBRT only; 110 (11.2%) received brachytherapy and EBRT; and 23 (2.3%) had unknown radiotherapy type.

^d^This group includes 16 hereditary and 4 nonhereditary patients with unknown chemotherapy.

^e^This group includes 1 hereditary and 3 nonhereditary patients with unknown radiotherapy.

^f^This group includes 8 hereditary and 13 nonhereditary patients with unknown radiotherapy and/or chemotherapy.

^g^Patients were followed from retinoblastoma diagnosis until the earliest of first incident subsequent malignant neoplasm or date of last contact (defined as the most recent questionnaire or, for patients who never completed a questionnaire, the earliest of death or estimated medical record abstraction date). Median follow-up to first SMN was 43.6 years (maximum = 67.6) among nonhereditary and 18.9 years (maximum = 56.6) years among hereditary survivors. Among survivors who did not develop an incident SMN during follow-up, nonhereditary patients had a median follow-up of 27.5 years (maximum = 89.3) and hereditary patients had a median follow-up of 23.9 years (maximum = 78.4 years).

There were 286 incident SMNs reported among 239/1128 (21.2%) hereditary patients (median time to first SMN = 18.9 years) and 28 incident SMNs among 25/924 (2.7%) nonhereditary patients (median time = 43.6 years; Tables 1 and 2; Supplementary Tables S2 and S3). Approximately 16% of hereditary survivors who developed an SMN had multiple SMNs compared with 8% among nonhereditary patients (Supplementary Tables S3 and S4). The maximum number of SMNs reported was 4—one hereditary patient developed a bladder SMN followed by three SMNs of the nasal cavity (Supplementary Tables S3 and S4).

**Table 2:** Risk for incident subsequent malignant neoplasms among hereditary and nonhereditary retinoblastoma survivors compared with that in the general population.

SMN type	Hereditary (*N* = 1128)						Nonhereditary (*N* = 924)					
	PY^a^	Obs	% Total	*N* known histology^b^	SIR (95% CI)^c^	AER^d^	PY^a^	Obs	% Total	*N* known histology^b^	SIR (95% CI)^c^	AER^d^
SMN total^e^	27,546	239 (265)^f^	100	224 (249)^f^	**11.9 (10.4–13.5)**	79.5	26,729	25 (27)^f^	100	23 (25)^f^	0.8 (0.5 –1.2)	−2.7
STS	28,466	89	34	89	**85.4 (68.6 –105.1)**	30.9	26,964	2	7	2	–	
Bone	28,569	80	30	77	**323.4 (256.4–402.5)**	27.9	26,980	0	0	NA	–	
CNS	28,901	6	2	5	**6.5 (2.4–14.2)**	1.8	26,972	1	4	1	–	
Breast	28,831	12	5	11	**3.1 (1.6–5.4)**	2.8	26,869	8	30	8	1.3 (0.6–2.5)	0.6
Female breast	13,830	12			**3.1 (1.6–5.5)**	5.9	13,811	8			1.3 (0.6–2.5)	1.3
Oral cavity^g^	28,840	5	2	3	**7.8 (2.5–18.1)**	1.5	26,980	0	0	NA	–	
Nasal cavity^h^	28,875	11	4	8	**297.7 (148.4–532.6)**	3.8	26,980	0	0	NA	–	
Uterine corpus	13,885	2	1	2	–		13,921	0	0	NA	–	
Kidney	28,888	2	1	2	–		26,969	1	4	1	–	
Gastrointestinal	28,913	1	0	1	–		26,965	3	11	3	0.9 (0.2–2.6)	−0.2
Liver/gallbladder	28,912	0	0	NA	–		26,980	0	0	NA	–	
Lung	28,911	3	1	3	1.9 (0.4–5.4)	0.5	26,966	2	7	1	–	
Cervix	13,911	0	0	NA	–		13,921	1	4	0	–	
Ovary	13,911	0	0	NA	–		13,921	0	0	NA	–	
Thyroid	28,858	3	1	2	2.4 (0.5–6.9)		26,942	2	7	2	–	
Bladder	28,901	1	0	1	–		26,974	1	4	1	–	
Melanoma	28,603	28	11	28	**17.0 (11.3–24.6)**	9.2	26,955	3	11	3	1.4 (0.3–4.1)	0.3
Pancreas	28,914	1	0	1	–		26,980	0	0	NA	–	
Prostate	15,001	0	0	NA	–		13,058	1	4	1	–	
Haematologic	28,873	6	2	5	1.8 (0.7–4)	0.9	26,972	1	4	1	–	
Pineoblastoma	28,910	8	3	8	**1930 (831.0–3803)**	2.8	26,980	0	0	NA	–	
Other/unspecified	28,891	7	3	3	**4.4 (1.8–9.0)**	1.9	26,974	1	4	1	–	

*SMN* subsequent malignant neoplasm, *STS* soft tissue sarcoma, *CNS* central nervous system, *Obs* observed number of SMNs, *PY* person-years, *SIR* standardised incidence ratio, *AER* absolute excess risk, *CI* confidence interval, *NA* not available.

^a^Patients were followed from retinoblastoma diagnosis until earliest of first incident subsequent malignant neoplasm of interest or date of last contact (defined as the most recent questionnaire or, for patients who never completed a questionnaire, the earliest of death or estimated medical record abstraction date).

^b^Number of cases with ICD-O-3 morphology codes excluding 8000–8001.

^c^SIR; observed/expected where the expected numbers are derived from SEER 9 rates 1975–2016 (with rates from 1975 to 1979 applied for earlier years), stratified by calendar year (1975–1979, 1980–1984, …, 2010–2016), sex and age (0–4, 5–9, …, 80–84, 85+), and multiplied by stratum-specific person-years at risk in the cohort. SIRs and AERs not shown when observed <3. Values in bold indicate statistically significant SIRs (corresponding to exclusion of 1.0 from the confidence limit).

^d^AER per 10,000 person-years ([(obs − expected)/person-years] × 10,000).

^e^SMN total includes all subsequent malignant neoplasms excluding retinoblastoma, orbit and nonmelanoma skin cancer.

^f^Number observed represents the number of individuals who developed any SMN (i.e. the first SMN). The number within parentheses represents the total number of SMNs, counting multiple SMNs per person but only the first occurrence of each type of SMN.

^g^Includes oral cavity and pharynx.

^h^Includes nasal cavity, middle ear and sinus.

Sarcomas of the soft tissue and bone contributed over 50% of SMNs among hereditary survivors, followed by melanoma, female breast, and nasal cavity (Table 2; Supplementary Table S5 shows SMNs by histology and topography). Compared with the general US population, hereditary survivors had a statistically significantly increased risk of developing an SMN (total: *N* = 239; SIR = 11.9; 95% confidence interval [CI] 10.4–13.5), corresponding to 79.5 excess cases per 10,000 person-years. The highest SIRs (>80-fold) were observed for sarcomas (soft tissue and bone), nasal cavity tumours and pineoblastoma. Lower but significantly increased risks were observed for melanoma and SMNs of the CNS, oral cavity and female breast (SIRs = 3.1–17). SIRs were not significantly elevated for epithelial malignancies of the uterine corpus, kidney, lung, bladder, pancreas or other types. Among nonhereditary survivors, risk was not statistically significantly elevated for SMNs overall (*N* = 25; SIR = 0.8; 95% CI 0.5–1.2); for female breast cancer, the most common SMN type among nonhereditary survivors (*N* = 8; SIR = 1.3; 95% CI 0.6–2.5); or for other SMN types evaluated.

**Fig. 1:**
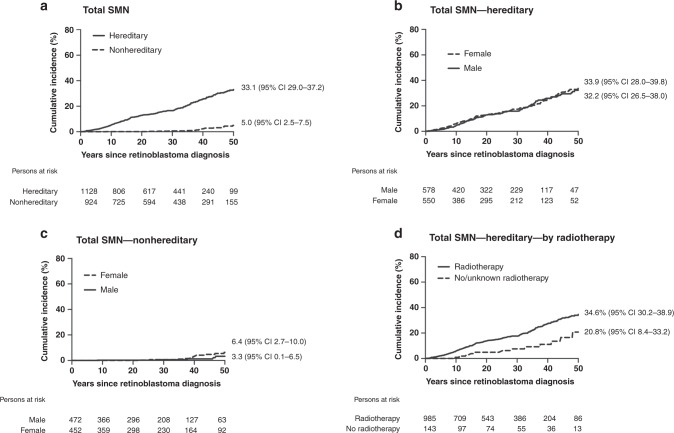
Cumulative incidence of developing a subsequent malignant neoplasm (SMN) up to 50 years following retinoblastoma diagnosis, accounting for competing risk of death^a^. **a** Cumulative incidence by hereditary retinoblastoma status. **b** Cumulative incidence by sex among hereditary retinoblastoma survivors. **c** Cumulative incidence by sex among nonhereditary retinoblastoma survivors. **d** Cumulative incidence by receipt of radiotherapy for retinoblastoma among hereditary survivors. ^a^Patients were followed from retinoblastoma diagnosis until diagnosis of first incident SMN or date of last contact (defined as the most recent questionnaire or, for patients who never completed a questionnaire, the earliest of death or estimated medical record abstraction date). Cumulative incidence at 50 years following retinoblastoma is presented in the figure. CI confidence interval.

The cumulative incidence of developing an SMN at 50 years following retinoblastoma diagnosis, accounting for competing risk of death, was 33.1% among hereditary (95% CI 29.0–37.2) and 5.0% among nonhereditary (95% CI 2.5–7.5) survivors (Fig. 1a). Among hereditary survivors, cumulative incidence began rising within the first few years after retinoblastoma diagnosis and continued to increase thereafter. Among nonhereditary survivors, there were few SMNs before 20 years and the most rapid increase was observed after 40 years. Irrespective of hereditary status, cumulative incidence at 50 years was similar among males and females (Fig. 1b, c). Lastly, cumulative incidence at 50 years was higher among hereditary survivors who received radiotherapy (34.6%; 95% CI 30.2–38.9) versus those who did not (20.8%; 95% CI 8.4–33.2).

**Fig. 2:**
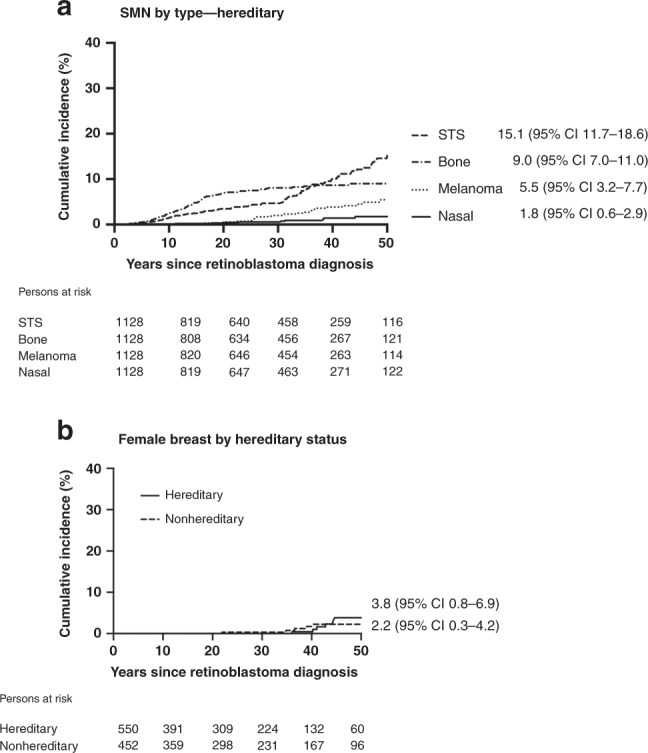
Type-specific cumulative incidence of developing a subsequent malignant neoplasm (SMN) up to 50 years following retinoblastoma diagnosis, accounting for competing risk of death^a^. **a** Type-specific cumulative incidence among all hereditary retinoblastoma survivors. **b** Cumulative incidence of breast cancer among female hereditary and nonhereditary retinoblastoma survivors. ^a^Patients were followed from retinoblastoma diagnosis until diagnosis of first incident SMN or date of last contact (defined as the most recent questionnaire or, for patients who never completed a questionnaire, the earliest of death or estimated medical record abstraction date). Cumulative incidence at 50 years following retinoblastoma is presented in the figure. CI confidence interval, STS soft tissue sarcoma. Nasal includes malignancies of the nasal cavity, sinus and middle ear.

The SMN types underlying the steady increase in the cumulative incidence among hereditary survivors varied by follow-up time (Fig. 2a). Subsequent bone and soft tissue sarcomas as well as nasal cavity cancers emerged within 5 years of retinoblastoma diagnosis. The cumulative incidence was highest for bone sarcomas until ~40 years, after which it was surpassed by soft tissue sarcoma, reaching cumulative incidences at 50 years of 15.1% and 9.0% for soft tissue and bone sarcoma, respectively. SMNs of the nasal cavity reached a cumulative incidence of 1.8% at 50 years. The cumulative incidence of melanoma began rising ~15 years and continued to increase, reaching 5.5% at 50 years. Subsequent female breast cancers appeared in the third decade after retinoblastoma diagnosis and increased thereafter, reaching 3.8% among hereditary and 2.2% among nonhereditary female survivors at 50 years (Fig. 2b).

**Fig. 3:**
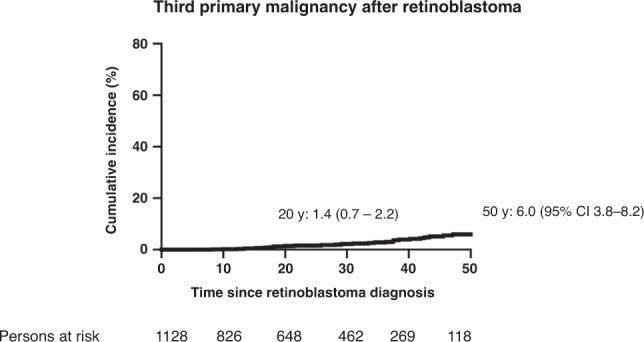
Cumulative incidence of developing an incident third primary malignancy (second SMN) accounting for competing risk of death among all hereditary retinoblastoma survivors^a^. ^a^Patients were followed from the date of retinoblastoma diagnosis until the diagnosis of incident second SMN (third primary malignancy) or last contact (defined as the most recent questionnaire, or for patients who never completed a questionnaire.

During follow-up, third primary malignancies (second SMNs) were reported among 38 hereditary survivors (median interval first-to-second SMN = 4.2 years; range 0–20.9). The cumulative incidence of developing a second SMN was 6.0% (95% CI 3.8–8.2) at 50 years (Fig. 3). Soft tissue sarcomas were most common (~34% of cases) followed by bone and melanoma (Supplementary Table S4). The majority of second SMNs (23/38) was of a different type than the first (Supplementary Tables S3 and S4). The cumulative incidence of developing a second SMN was 28.1% (95% CI 19.3–37.0) at 20 years following the first SMN.

Among hereditary survivors, earlier age and year of retinoblastoma diagnosis were associated with increased risk of total SMN; similar albeit nonstatistically significant patterns were observed for SMNs of the soft tissue, bone, breast (calendar year only), nasal cavity and melanoma (Supplementary Table S6). Family history of retinoblastoma was positively associated with total SMN risk; although not statistically significant, similar increases also were observed for SMNs of the breast, nasal cavity and melanoma. Compared with patients who received radiotherapy only, the risks for total SMN as well as SMNs of the soft tissue, bone and breast were nonsignificantly higher among patients who also received chemotherapy (Supplementary Table S6). Unlike other SMN types, breast cancer risk appeared higher among patients who had not received cytotoxic therapy, albeit based on small numbers (Supplementary Tables S6 and S7). None of the factors examined were significantly associated with incident SMNs (total) among nonhereditary survivors (Supplementary Table S8).

In sensitivity analyses, the inclusion of mortality-based cases generally increased the SIRs among hereditary survivors (Supplementary Table S9). Notably, unlike the main incident analyses, the SIRs were statistically significantly elevated for SMNs of the uterine corpus, kidney, bladder, lung and pancreas. The corresponding cumulative incidence was also generally higher (e.g. cumulative incidence for total SMN at 50 years after hereditary retinoblastoma = 44.1%; 95% CI 40.1–48.0). Analyses restricted to second primary incident cancers yielded similar SIRs and cumulative incidence estimates to our main analyses (data not shown).

## Discussion

In this large-scale, comprehensive investigation of SMNs after retinoblastoma leveraging detailed SMN diagnostic data with histologic information, we demonstrate an increased risk among hereditary retinoblastoma survivors for incident SMNs of the CNS, nasal cavity, oral cavity and breast, and confirm well-established substantially elevated risks for bone and soft tissue sarcoma (>80-fold), melanoma (17-fold) and pineoblastoma (>100-fold). However, we did not observe a significantly increased risk for other epithelial malignancies, including kidney, bladder, uterus, pancreas or lung. The cumulative incidence of second (33.1%) and third primary (6.0%) malignancies at 50 years following retinoblastoma emphasise the substantial long-term burden of SMNs in this population. In contrast to hereditary survivors, incident SMN rates among nonhereditary survivors were comparable to those in the general population. Markedly lower risks among nonhereditary survivors compared with hereditary survivors likely reflect both differences in underlying genetic susceptibility to SMNs as well as treatment differences. Nonhereditary patients in our study were all diagnosed with unilateral retinoblastoma, which was primarily treated with enucleation of the affected eye.^20^

### Burden of SMNs

With a median follow-up of 22.6 years and 10% of the population having reached ages ≥50 years at first SMN or exit, our results confirm that the burden of SMNs among hereditary retinoblastoma survivors is largely driven by soft tissue sarcomas and bone tumours followed by melanoma. Together, these malignancies accounted for over 70% of SMNs among hereditary patients, with corresponding cumulative incidence estimates at 50 years of 15.1%, 9%, and 5.5%, respectively. The patterns, however, may be important when considering potential surveillance strategies. Whereas bone and soft tissue sarcomas were first observed within 5 years of retinoblastoma, few melanomas were observed before the age of 15 years. With respect to risk factors for these most common SMNs, we observed higher risks for bone and soft tissue sarcoma for individuals diagnosed with retinoblastoma at younger ages, before 1960, and who received initial radiotherapy/chemotherapy. The association with age may indicate a role of highly penetrant *RB1* mutations in the risk of SMN, although at present time there is only preliminary evidence for an association between mutation penetrance and SMN,^21,22^ while the calendar year findings may reflect decreases in radiation dose over time.^7,10,20^ For melanoma, only a family history of retinoblastoma appeared to be associated with risk. Although none of these associations were statistically significant in our incident-based analyses, they are consistent with previously reported patterns from our cohort^3,10^ and elsewhere.^1^

This is the first study, to our knowledge, to quantify the cumulative incidence for developing a third primary cancer (second SMN) after hereditary retinoblastoma. We estimate that ~6% of all hereditary survivors developed a third primary malignancy by the age of 50 years, while 28% of survivors who already had a second primary cancer developed another one within 20 years of the first, most commonly of a different type. These findings complement a previous study from the Netherlands that reported a markedly elevated risk for developing a new primary malignancy, based on 11 cases, among survivors of a second primary malignancy, compared with both the general population and retinoblastoma survivors who had not developed a second malignancy.^14^

### Epithelial cancers

Previous studies (including our own mortality study^20^) have suggested wide-ranging increased risks for epithelial tumours both in (head/neck) and out (body/limbs) of the radiation field^1,2,8^ among hereditary retinoblastoma survivors. For a number of sites including bladder, uterus, kidney, lung and pancreas, we only observed increased risk when mortality cases (the majority of which lack histologic information) were included. For bladder, uterus and kidney cancers, we hypothesise that misclassified sarcomas included with the mortality cases may explain the discrepancies between our primary analyses and both the sensitivity analysis and previous reports in which we and others included mortality-based cases. In particular, there is a clear, increased risk for uterine leiomyosarcoma after hereditary retinoblastoma.^23^ Uterine sarcomas accounted for the majority of uterine cancers in previous analyses in our cohort^5^ and another large UK cohort that included 806 hereditary retinoblastoma survivors,^2^ which both reported elevated risks of uterine SMNs. Similarly, sarcomas accounted for 6/8 bladder cancers underlying the increased risk reported in the UK study.^2^ Lastly, our incident-based analysis does not provide any evidence for a risk of epithelial tumours of the kidney or gastrointestinal tract after hereditary retinoblastoma. Increased risk for cancers of the kidney and large intestine were observed in our recent mortality study, but notably sarcomas accounted for at least one of the three kidney cancer deaths and two of the four large intestine cancer deaths.^20^

In contrast to the sites above, we continue to report significantly increased risks for tumours of the nasal cavity, oral cavity, CNS and breast, as well as pineoblastoma. Receipt of radiotherapy for retinoblastoma likely explains the substantially increased risk observed for tumours in or near the radiation field—nasal and oral cavity, CNS and potentially pineoblastoma. Notably, all of these SMNs occurred among survivors who had received radiotherapy. The availability of histologic data in this study reduces the potential for misclassified sarcomas or metastatic retinoblastoma to explain the observed risks of nasal and oral cavity SMNs. Although we cannot preclude the possibility that CNS tumours were misclassified metastatic retinoblastoma or pineoblastoma, the latency pattern (5/6 CNS SMNs diagnosed ≥9 years after retinoblastoma) would be consistent with radiation-induced tumours^24^ and is longer than that the generally reported for pineoblastoma.^15^

The 3-fold increased risk of breast cancer we observed based on 12 cases among hereditary survivors is consistent with our previous reports^5,19^ as well as the Dutch and UK cohorts, although only statistically significant in the latter and based on fewer (United Kingdom: 8; Dutch: 2) cases than we observed.^1,2^ Because risks were significantly higher among hereditary retinoblastoma survivors without known radiation or chemotherapy than those with radiotherapy, we think that radiotherapy is unlikely to explain the observed increase. Our data provide some evidence, however, for an association with chemotherapy; although not statistically significant, we observed a nearly 5-fold increased risk among females who had received chemotherapy in addition to radiotherapy. The SIR among nonhereditary survivors (1.3) was lower than our previous estimate (2.8) and no longer statistically significant. This null finding is consistent with the Dutch cohort, the only other study (to our knowledge) to have reported an SIR for breast cancer among nonhereditary patients.^1^ Further research is needed to explain whether chemotherapy or some other factors, including hormonal and reproductive, explain the weakly increased breast cancer risk after hereditary retinoblastoma compared with the general population. The markedly lower risk of breast compared with other malignancies such as sarcomas and melanoma, as well as the higher risk observed among survivors diagnosed at ages 12–23 months versus <12 months (albeit based on small numbers) may suggest less of a role for genetic susceptibility.

Cancers of the lung and pancreas are often diagnosed at advanced stages, which is associated with poorer survival,^9^ and thus it is likely that the method of ascertaining incident cases (medical records and patient report) led to an underestimate of risk compared with the general population. This may explain why we observed risks for these cancers only when we included mortality-based cases. A number of earlier reports showed increased lung cancer risk after hereditary retinoblastoma,^1,2,5,8^ but most of those studies included SMNs ascertained from death certificates.

Strengths of this study include long-term follow-up, the large number of SMNs and detailed histologic information from the incident cases. Compared with the previous comprehensive overview of SMN risk in the cohort,^5^ the present study includes up to 16 more years of follow-up for individuals diagnosed with retinoblastoma during 1914–1984 and expands the analyses to include patients diagnosed from 1985 to 2006. A primary limitation of these data is our method of ascertaining subsequent cancers (medical record review at the time of cohort initiation and self-report), which likely led to an under-ascertainment of cases, in part from nonresponse to follow-up questionnaires. As such, the true SIRs and cumulative incidence may be higher than those reported here. Inclusion of mortality cases captures some additional cases, but that comes at a loss of histologic information that increases the chances for the inclusion of sarcomas with site-specific cancers. While our earlier assessment of potential bias based on nonresponse to questionnaires was generally reassuring given similarities between responders and nonresponders with respect to baseline characteristics and mortality,^10^ as is the case for any cohort study that relies in part on self-report of outcomes, we cannot rule out the potential for bias that could arise if SMN risk differs by the availability of follow-up information. Although we noted long-term follow-up data as a strength of this analysis, there were limited numbers of survivors remaining in the analysis 50 years after retinoblastoma diagnosis. This limitation is particularly pertinent to breast, lung and other epithelial malignancies, which are more likely to be diagnosed at older ages. Linkage to cancer registry data would improve long-term cancer ascertainment, but at present time the United States does not currently have a national cancer registry. We sought to address the limitation of the lack of treatment data for subsequent malignancies by conducting sensitivity analyses in which follow-up was censored at diagnosis of a second primary malignancy. It is reassuring that our overall conclusions did not materially change with this approach. Importantly, given the calendar years of retinoblastoma diagnosis in our cohort, our results may not be generalisable to hereditary survivors diagnosed more recently. Specifically, the majority of patients in our study were treated largely with external beam radiotherapy, with the highest doses received by patients treated prior to 1960,^7^ with or without systemic chemotherapy. Thus, we could not investigate SMN risks associated with brachytherapy. Because the majority of our cohort was diagnosed before 2000, we also could not evaluate risks associated with localised (i.e. intravitreal and intra-arterial) chemotherapy.^25,26^ In addition, as the majority of hereditary patients received radiotherapy, we have limited information about SMN risks in the absence of radiotherapy. A strength of this study was the relatively small proportion of patients for whom receipt of radiotherapy or chemotherapy was unknown (~2%). We included the unknown with those not receiving the treatment; while this may have led to some under-ascertainment of associations between treatment and SMN risk, we did not have an adequate sample size to classify the unknown groups separately. Lastly, in the absence of *RB1* germline mutation data, we classified hereditary status based on laterality and family history. In doing so, we may have misclassified some hereditary cases as having nonhereditary disease.

## Conclusion

In conclusion, our results confirm substantially elevated risks for bone and soft tissue sarcomas and melanoma, but suggest that the risk for epithelial cancers among hereditary retinoblastoma survivors is less wide-ranging than previously suggested. We demonstrate an increased risk in this population for incident SMNs of the breast, CNS, nasal cavity, oral cavity and pineoblastoma, but did not observe significantly increased risks for other epithelial malignancies, including kidney, bladder, uterus, pancreas or lung. The cumulative incidence estimates, including that for developing a second SMN, emphasise the substantial long-term burden of SMNs in these survivors. The notable variation in the incidence patterns across SMN types should be considered when developing surveillance strategies.

## Supplementary information

Supplemental Material

## Data Availability

All anonymised data used in the paper can be provided upon request upon application to the corresponding author.
