# Supplementary material for: Long-term risk of subsequent cancer incidence among hereditary and nonhereditary retinoblastoma survivors
Source: Br J Cancer. 2021 Jan 21;124(7):1312–9. doi: 10.1038/s41416-020-01248-y (PMC8007574; doi:10.1038/s41416-020-01248-y)
Supplement: Supplementary file 1 — Supplemental Material [file 41416_2020_1248_MOESM1_ESM.pdf]

Supplementary Table S1. Classification of incident subsequent malignant neoplasms

**Subsequent malignant neoplasm type**	**Classification**
Bone and soft tissue sarcomas	See Appendix Table A2 in Kleinerman et al. 2019[a] which is based on a modified version of International Classification of Childhood Cancers[b]
All other SMNs (excluding bone and soft tissue sarcomas as defined above)	Based on SEER Site Recode (unless otherwise specified)<br><br>(https://seer.cancer.gov/siterecode/icdo3_dwhoheme/)
Central Nervous System	31010, 31040
Breast	26000
Oral cavity/pharynx	20010-20100
Nasal cavity/middle ear/sinus	22010
Uterine corpus	27020, 27030
Kidney	29020
Gastrointestinal	21010-21060
Liver/gallbladder	21071-21090
Lung	22030
Cervix	27010
Ovary	27040
Thyroid	32010
Bladder	29010
Melanoma	25010
Pancreas	21100
Prostate	28010
Hematologic	33011-35043
Pineoblastoma	ICD-O-3 morphology: 9362
Other/unspecified	All other malignancies excluding retinoblastoma (ICD-O-3 morphology: 9510-9514; ICD-O-3 topography: C69.2), orbit (ICD-O-3 topography: C69.6) and non-melanoma skin cancer (topography: C44.0-C44.9 and morphology codes excluding 8720-8790)

ICD-O=International Classification of Diseases for Oncology; SEER=Surveillance, Epidemiology, and End Results Program

a. Kleinerman RA, Schonfeld SJ, Sigel BS, Wong-Siegel JR, Gilbert ES, Abramson DH, et al. Bone and Soft-Tissue Sarcoma Risk in Long-Term Survivors of Hereditary Retinoblastoma Treated with Radiation. J Clin Oncol 2019;37(35):3436-45
b. Steliarova-Foucher E, Stiller C, Lacour B, et al. International Classification of Childhood Cancer, third edition. Cancer. 2005;103(7):1457–1467

Supplementary Table S2. Interval (years) between diagnosis of retinoblastoma and second malignant neoplasms (SMN), by hereditary status[a] and SMN type.

	Nonhereditary[b]			Hereditary[b]		
	N	Median interval (y)	Range (y)	N	Median interval (y)	Range (y)
SMN Total	25	43.6	(8.5-67.6)	239	18.9	(0-56.6)
STS	2	48.4	(46.9-49.9)	89	31.0	(2-56.6)
Bone	0			80	13.5	(1.5-43.4)
CNS	1	8.5		6	12.1	(0-40.3)
Breast	8	39.7	(22-57.6)	12	48.0	(36.1-55.4)
Oral cavity[c]	0			5	19.0	(4.3-42.1)
Nasal cavity[d]	0			11	30.6	(5.5-64)
Uterine corpus	0			2	35.5	(28.5-42.6)
Kidney	1	47.1		2	17.7	(0.9-34.5)
Gastrointestinal	3	43.6	(39.7-56.3)	1	43.3	
Liver/gallbladder	0			0		
Lung	2	64.7	(61.9-67.6)	3	37.6	(35.6-38.5)
Cervix	1	57.5		0		
Ovary	0			0		
Thyroid	2	32.1	(25.5-38.7)	3	19.0	(15.8-58.9)
Bladder	1	40.7		1	56.0	
Melanoma	3	52.6	(37.8-53.8)	28	28.7	(14.1-48.9)
Pancreas	0			1	54.5	
Prostate	1	60.5		0		
Hematologic	1	46.3		6	16.0	(3.8-39.5)
Pineoblastoma	0			8	2.4	(1.3-8.8)
Other/unspecified	1	29.0		7	10.9	(1.9-36.1)

STS = soft tissue sarcoma; CNS = central nervous system

a. Hereditary status was determined based on medical records at the time of retinoblastoma diagnosis. Patients were classified as having hereditary retinoblastoma if they had bilateral retinoblastoma or unilateral disease and a family history of retinoblastoma. Individuals with unilateral retinoblastoma and no known family history at the time of retinoblastoma diagnosis were classified as having non-hereditary disease.
b. Number, median and range based on the first occurrence of each SMN type.
c. Includes oral cavity and pharynx
d. Includes nasal cavity, middle ear and sinus.

Supplementary Table S3. Count of subsequent malignant neoplasms (SMNs) by hereditary status[a] among individuals with at least one SMN.

	Nonhereditary		Hereditary	
	N	%	N	%
Number of SMNs[b]				
1	23	92.0%	201	84.1%
2	1	4.0%	30	12.6%
3	1	4.0%	7	2.9%
4	0	0.0%	1	0.4%
Types of SMNs[c]				
1	24	96.0%	214	89.5%
2	0	0.0%	24	10.0%
3	1	4.0%	1	0.4%
Agreement first and second SMNs[d]				
Only one SMN	23	92.0%	201	84.1%
Same type	1	4.0%	15	6.3%
Different types	1	4.0%	23	9.6%

a. Hereditary status was determined based on medical records at the time of retinoblastoma diagnosis. Patients were classified as having hereditary retinoblastoma if they had bilateral retinoblastoma or unilateral disease and a family history of retinoblastoma. Individuals with unilateral retinoblastoma and no known family history at the time of retinoblastoma diagnosis were classified as having non-hereditary disease.
b. Including multiple SMNs of the same type (e.g., an individual with two subsequent melanomas and no other SMNs was counted as having two SMNs).
c. Counting only the first occurrence of each SMN type (e.g., an individual with two subsequent melanomas and no other SMNs was counted as having one type).
d. Comparison of SMN 1 and SMN 2. An individual with two subsequent melanomas followed by a soft tissue sarcoma was classified as “same type” for this variable.

Supplementary Table S4. Subsequent malignant neoplasm (SMN) combinations by sequence among 239 hereditary retinoblastoma survivors with at least one SMN.

First SMN	Second SMN	Third SMN	Fourth SMN	Fifth SMN	*N*
**STS**	.	.	.	.	70
	**STS**	.	.	.	5
	**Bone**	.	.	.	2
	**Uterine corpus**	**Thyroid**	.	.	1
	**Kidney**	.	.	.	1
**Bone**	.	.	.	.	64
	**STS**	.	.	.	4
		**STS**	.	.	1
	**Bone**	.	.	.	3
		**STS**	.	.	1
	**Nasal cavity/middle ear/sinus**	.	.	.	1
	**Melanoma**	**Melanoma**	.	.	1
**CNS**	.	.	.	.	4
	**Bone**	.	.	.	1
**Breast**	.	.	.	.	7
	**Breast**	.	.	.	2
	**Melanoma**	.	.	.	1
**Oral cavity/pharynx**	.	.	.	.	4
	**Melanoma**	**Melanoma**	.	.	1
**Nasal cavity/middle ear/sinus**	.	.	.	.	8
	**Bone**	.	.	.	1
**Uterine corpus**	.	.	.	.	1
**Kidney**	.	.	.	.	1
**GI**	**STS**	.	.	.	1
**Lung**	.	.	.	.	3
**Thyroid**	.	.	.	.	2

**Bladder**	**Nasal cavity/middle ear/sinus**	**Nasal cavity/middle ear/sinus**	**Nasal cavity/middle ear/sinus**	.	1
**Melanoma**	.	.	.	.	17
	**STS**	.	.	.	2
	**Breast**	.	.	.	2
	**Melanoma**	.	.	.	2
		**STS**	.	.	1
		**Melanoma**	.	.	1
**Pancreas**	.	.	.	.	1
**Hematologic**	.	.	.	.	5
	**Bone**	.	.	.	1
**Other/unspecified**	.	.	.	.	6
	**CNS**	.	.	.	1
**Pineoblastoma**	.	.	.	.	8

SMN=subsequent malignant neoplasm; STS=soft tissue sarcoma; CNS=central nervous system, GI=gastrointestinal;

Supplementary Table S5. Observed subsequent malignant neoplasms (SMNs)[a] by histology and location

SMN Type	By morphology (observed codes)		Observed topography codes	Hereditary (N)	Nonhereditary (N)
Soft tissue sarcoma	Fibrosarcoma, malignant fibrous histiocytoma (ICD-O: 8810, 8830)	Nasal cavity, sinus, nasopharynx, parotid	ICD-9: 160.0, 160.3, 160.4; ICD-O: C31.0, C31.1, C31.3	9	
		head, brain, face, eyelid, lip	ICD-9: 171.0, 173.1, 192.1; ICD-O: C44.1, C49.0	7	
		Orbit	ICD-9: 190.1	6	
		Thorax, trunk, abdomen, upper limb, lower limb	ICD-9: 173.6; ICD-O: C49.1	2	
	Liposarcoma (ICD-O: 8850-8852)	Head, brain, face, eyelid, lip	ICD-O: C49.0	2	
		Thorax, trunk, abdomen, upper limb, lower limb	ICD-9: 171.4	1	
		Uterus, pelvis, retroperitoneum, scrotum	ICD-9: 171.6	1	
	Leiomyosarcoma (ICD-O: 8890-8891, 8896)	Nasal cavity, sinus, nasopharynx, parotid	ICD-9: 160.0, 160.2; ICD-O: C11.9, C30.0, C31.0, C31.3	8	
		Head, brain, face, eyelid, lip	ICD-O: C49.0	1	
		Orbit	ICD-9: 190.1; ICD-O: C69.6	4	
		Thorax, trunk, abdomen, upper limb, lower limb	ICD-9: 173.5; ICD-O: C38.1, C49.1, C49.2, C49.4, C76.2	10	
		Uterus, pelvis, retroperitoneum, scrotum	ICD-9: 158.-, 171.6, 179.9, 182.0, 187.7; ICD-O: C48.0, C49.5, C54.2, C55.9	11	2
		Kidney, bladder, colon (cecum)	ICD-9: 153.4; ICD-O: C18.0, C18.7	3	
	Rhabdomyosarcoma (ICD-O: 8900-8901, 8910, 8920)	Nasal cavity, sinus, nasopharynx, parotid	ICD-9: 160.0	1	
		Head, brain, face, eyelid, lip	ICD-9: 171.0, 191.9; ICD-O: C49.0	10	
	Other (ICD-O: 8940, 8950, 8980, 8990, 9040, 9120, 9560)	Nasal cavity, sinus, nasopharynx, parotid	ICD-9: 142.0, 160.2	2	
		Head, brain, face, eyelid, lip	ICD-9: 171.0; ICD-O: C00.3, C49.0	3	
		Orbit	ICD-9: 190.1	1	
		Thorax, trunk, abdomen, upper limb, lower limb	ICD-9: 171.4	1	
		Uterus, pelvis, retroperitoneum, scrotum	ICD-O: 49.5, 55.9	2	
		Unknown	ICD-9: 199.9	1	
	Unknown (ICD-O: 8800, 8802, 8805)	Nasal cavity, sinus, nasopharynx, parotid	ICD-9: 160.0; ICD-O: C31.0	2	
		Orbit	ICD-O: C69.6	1	

Bone	Malignant fibrous histiocytoma, spindle cell sarcoma (ICD-O: 8810)	Skull and face, hard palate	ICD-9: 170.0	1	
	Osteosarcoma (ICD-O: 9180-9183, 9186, 9250)	Skull and face, hard palate	ICD-9: 145.2, 170.0; ICD-O: C41.0, C41.1	37	
		Nasal cavity, sinus	ICD-9: 160.0, 160.3; ICD-O: C30.0, C31.0	6	
		Orbit	ICD-O: C69.6	1	
		Ribs/long bones upper limb, scapula and associated joints	ICD-9: 170.3, 170.4; ICD-O: C40.0	6	
		Long bones of lower limb and associated joints	ICD-9: 170.7; ICD-O: C40.2	23	
		Unknown	ICD-9: 170.9	1	
	Ewing sarcoma (ICD-O: 9260)	Long bones of lower limb and associated joints	ICD-9: 170.7	2	
	Unknown (Missing ICD-O)	Skull and face, hard palate	ICD-9: 170.0	1	
		Unknown		2	
Melanoma	Malignant melanoma, NOS (ICD-O: 8720)	Head/face/neck	ICD-9: 173.3, 173.4; ICD-O: C44.2, C44.3	6	
		Trunk, upper limb/shoulder, lower limb/hip	ICD-9: 173.5-173.7; ICD-O: C44.6	12	2
		Unknown	ICD-O: C44.9	2	
	Epithelioid cell melanoma (ICD-O: 8771)	Trunk, upper limb/shoulder, lower limb/hip	ICD-O: C44.7		1
	Nodular melanoma (ICD-O: 8721)	Head, face, neck	ICD-O: C44.3	1	
	Superficial spreading melanoma (ICD-O: 8743)	Head, face, neck	ICD-9: 173.3, ICD-O: C44.3, C44.4	3	
		Trunk, upper limb/shoulder, lower limb/hip	ICD-9: 173.5, ICD-O: C44.6	4	
		Unknown			
Central nervous system	Medulloblastoma (ICD-O: 9470)	Ventricles	ICD-9: 191.5	1	1
		Cerebrum	ICD-O: C71.0		
	Glioma (ICD-O: 9380)	Frontal lobe	ICD-9: 191.1	1	
	Glioblastoma (ICD-O: 9440)	Brain, NOS	ICD-9: 191.9	1	
	Meningioma (ICD-O: 9530)	Cerebral meninges, Meninges NOS	ICD-9: 192.1; ICD-O: C70.9	2	
	Unknown (ICD-O: 8000)	Nervous system, NOS	ICD-9: 192.9	1	
Breast	Infiltrating duct carcinoma, NOS (ICD-O: 8500)			6	6

	Infiltrating duct carcinoma mixed with other types of carcinoma (ICD-O: 8523)			1	
	Lobular carcinoma, NOS (ICD-O:8520)			2	1
	Small cell carcinoma (ICD-O: 8041)			1	
	Neuroendocrine carcinoma, NOS (ICD-O: 8246)			1	
	Carcinoma, NOS (ICD-O: 8010)				1
	Unknown (ICD-O: 8000)			1	
Oral cavity/pharynx	Squamous cell carcinoma (ICD-O: 8070)	Tongue	ICD-O: C02.9	1	
	Oxyphilic adenocarcinoma (ICD-O: 8290)	Parotid gland	ICD-9: 142.0	1	
	Carcinoma, NOS (ICD-O: 8010)	Tongue	ICD-9: 141.9	1	
	Unknown (ICD-O: 8000)	Head, face and neck, NOS	ICD-9: 195.0	2	
Nasal cavity, middle ear, sinus	Small cell carcinoma, NOS (ICD-O: 8041)	Sinus	ICD-O: C31.1	1	
	Squamous cell carcinoma, NOS (ICD-O: 8070)	Nasal cavity	ICD-9: 160.0	2	
		Sinus	ICD-9: 160.3	1	
	Adenocarcinoma, NOS (ICD-O: 8140)	Sinus	ICD-9: 160.3	1	
	Adenocarcinoma, intestinal type (ICD-O: 8144)	Nasal cavity	ICD-O: C30.0	1	
	Adenosquamous carcinoma (ICD-O: 8560)	Sinus	ICD-9: 160.3	1	
	Carcinoma, NOS (ICD-O: 8010)	Sinus	ICD-O: C31.1	1	
	Unknown (ICD-O: 8000 or missing)	Nasal cavity	ICD-9: 160.0	1	
		Sinus	ICD-9: 160.3	1	
		Missing	Missing	1	
Uterine corpus	Adenocarcinoma, NOS (ICD-O: 8140)			2	
Kidney	Papillary adenocarcinoma (ICD-O: 8260)				1
	Renal cell carcinoma (ICD-O: 8312)			1	
	Nephroblastoma (ICD-O: 8960)			1	
Gastrointestinal	Adenocarcinoma, NOS (ICD-O: 8140)	Rectosigmoid junction	ICD-9: 154.0		1

		Colon	ICD-9: 153.2; ICD-O: C18.2	1	1
	Adenocarcinoma in adenomatous polyp (ICD-O: 8210)	Colon	ICD-O: C18.4		1
Lung	Oat cell carcinoma (ICD-O: 8042)				1
	Small cell carcinoma, NOS (ICD-O: 8041)			1	
	Carcinoma, undifferentiated, NOS (ICD-O: 8020)			2	
	Unknown (ICD-O: 8000)				1
Cervix	Unknown (ICD-O: 8000)				1
Thyroid	Papillary carcinoma, follicular variant (ICD-O: 8340)				1
	Medullary carcinoma, NOS (ICD-O: 8510)				1
	Carcinoma, NOS (ICD-O: 8010)			1	
	Follicular carcinoma (ICD-O: 8335)			1	
	Unknown (ICD-O: 8000)			1	
Bladder	Transitional cell carcinoma (ICD-O: 8120)			1	1
Pancreas	Adenocarcinoma, NOS (ICD-O: 8140)			1	
Prostate	Adenocarcinoma, NOS (ICD-O: 8140)				1
Hematologic	Non-Hodgkin lymphoma (ICD-O: 9695)				1
	Hodgkin lymphoma (ICD-O: 9656, 9661)			2	
	Lymphoid leukemia, NOS (ICD-O: 9820)			1	
	Acute lymphoid leukemia (ICD-O: 9821)			1	
	Chronic lymphocytic leukemia (ICD-O: 9823)			1	
	Unknown (ICD-O: 8000)			1	
Pineoblastoma	Pineoblastoma (ICD-O: 9362)			8	

Other/unspecified	Seminoma, NOS (ICD-O: 9061)	Mediastinum	ICD-O: C38.3		1
	Neuroblastoma, NOS (ICD-O: 9500)	Head, face, neck	ICD-9: 171.0	1	
	Olfactory neuroblastoma (ICD-O: 9522)	Sinus	ICD-9: 160.3	1	
	Unknown (ICD-O: 8000 or missing)	Unknown	ICD-9: 199.9 or missing	5	

ICD: International Classification of Diseases; ICD-O: International Classification of Diseases for Oncology

a. Counting only the first occurrence of each SMN type.

Supplementary Table S6. Risk factors for subsequent malignant neoplasms among 1128 hereditary retinoblastoma survivors, overall and for types with at least 10 cases

Factors	SMN total[a]		Soft tissue sarcoma		Bone		Female breast		Nasal cavity		Melanoma	
	N	HR (95% CI)[b]	*N*	HR (95% CI)[b]	*N*	HR (95% CI)[b]	*N*	HR (95% CI)[b]	*N*	HR (95% CI)[b]	*N*	HR (95% CI)[b]
Total	*239*		*89*		*80*		*12*		*11*		*28*	
Age at retinoblastoma diagnosis												
<12 months	*152*	1.0 (REF)	*52*	1.0 (REF)	*53*	1.0 (REF)	*5*	1.0 (REF)	*7*	1.0 (REF)	*16*	1.0 (REF)
12-23 months	*54*	0.6 (0.5-0.9)	*25*	0.8 (0.5-1.3)	*15*	0.6 (0.3-1.0)	*6*	4.1 (1.0-16.7)	*2*	0.5 (0.1-2.5)	*8*	0.8 (0.4 - 2.0)
24+ months	*33*	0.8 (0.5-1.1)	*12*	0.7 (0.4-1.4)	*12*	0.9 (0.5-1.6)	*1*	0.8 (0.8-7.1)	*2*	1.2 (0.2-6.1)	*4*	0.8 (0.3 - 2.5)
P-trend[c]		***0.02***		*0.24*		*0.27*		*0.64*		*0.91*		*0.65*
Year of retinoblastoma diagnosis												
<1960	*98*	1.0 (REF)	*37*	1.0 (REF)	*23*	1.0 (REF)	*9*	1.0 (REF)	*6*	1.0 (REF)	*14*	1.0 (REF)
1960-1969	*71*	0.6 (0.5-0.9)	*28*	0.7 (0.4-1.1)	*27*	0.9 (0.5 - 1.6)	*3*	0.6 (0.1-2.5)	*3*	0.4 (0.1-1.7)	*8*	0.4 (0.2 - 1.0)
1970-2006	*70*	0.7 (0.5-0.9)	*24*	0.9 (0.5-1.5)	*30*	0.8 (0.5 - 1.5)	*0*		*2*	0.2 (0.04-1.4)	*6*	0.5 (0.2 - 1.3)
P-trend[c]		***0.01***		*0.47*		*0.51*		*0.29*		*0.09*		*0.08*
Sex												
Male	*118*	1.0 (REF)	*49*	1.0 (REF)	*41*	1.0 (REF)			*7*	1.0 (REF)	*13*	1.0 (REF)
Female	*121*	1.1 (0.8-1.4)	*40*	0.8 (0.5-1.2)	*39*	1.0 (0.7-1.6)	*12*	NA	*4*	0.6 (0.2-2.2)	*15*	1.2 (0.6 - 2.6)
P-value[d]		*0.67*		*0.31*		*0.90*				*0.47*		*0.60*
Family history of retinoblastoma												
No/unknown	*183*	1.0 (REF)	*76*	1.0 (REF)	*62*	1.0 (REF)	*9*	1.0 (REF)	*8*	1.0 (REF)	*20*	1.0 (REF)
Yes	*56*	1.4 (1.0-1.9)	*13*	0.8 (0.4-1.4)	*18*	1.2 (0.7-2.0)	*3*	4.4 (1.0-19.7)	*3*	1.9 (0.5-7.5)	*8*	2.2 (0.9 – 5.0)
P-value[d]		***0.04***		*0.39*		*0.59*		*0.06*		*0.35*		*0.07*
Treatment for retinoblastoma												
RT, no/uk CT	*119*	1.0 (REF)	*41*	1.0 (REF)	*40*	1.0 (REF)	*2*	1.0 (REF)	*9*	1.0 (REF)	*15*	1.0 (REF)
RT and CT	*106*	1.2 (0.9-1.6)	*44*	1.4 (0.9-2.3)	*36*	1.3 (0.8-2.1)	*7*	4.8 (1.0-23.7)	*2*	0.3 (0.1-1.3)	*12*	1.0 (0.5 - 2.2)
CT, no/uk RT	*1*	0.2 (0.03-1.4)	*0*	NA	*1*	0.6 (0.1-4.1)	*0*	NA	*0*	NA	*0*	NA
No/uk RT or CT	*13*	0.5 (0.3-0. 9)	*4*	0.4 (0.2-1.3)	*3*	0.4 (0.1-1.4)	*3*	8.3 (1.2-58.1)	*0*	NA	*1*	0.3 (0.03 - 2.0)
P-value[d]		***0.01***		*0.09*		*0.18*		*0.17*		*0.47*		*0.64*

SMN=subsequent malignant neoplasm; HR=hazard ratio; CI=confidence interval; CT=chemotherapy; RT=radiotherapy; uk=unknown;

a. SMN total includes all subsequent malignant neoplasms excluding retinoblastoma, orbit and non-melanoma skin cancer.

b. Hazard ratios (HRs) and 95% confidence intervals (CI) were estimated from Cox proportional hazards models, mutually adjusted for factors in the table. Patients were followed from retinoblastoma diagnosis until earliest of first incident subsequent malignant neoplasm of interest or date of last contact (defined as the most recent questionnaire or, for patients who never completed a questionnaire, the earliest of death or estimated medical record abstraction date).
c. Wald P-value was obtained by treating the ordinal variable as a continuous variable (with all other variables modelled categorically).
d. Wald P-value for the categorical variable in the model (with all variables modelled categorically).

Supplementary Table S7. Risk for incident subsequent malignant neoplasms among hereditary retinoblastoma survivors compared with that in the general population, by treatment for retinoblastoma.

	RT, no/uk CT			RT and CT			CT, no/uk RT			No/uk RT or CT		
SMN Type	PY[a]	Obs	SIR (95% CI)[b]	PY[a]	Obs	SIR (95% CI)[b]	PY[a]	Obs	SIR (95% CI)[b]	PY[a]	Obs	SIR (95% CI)[b]
SMN Total[c]	14022	119	**12.1 (10.0 - 14.5)**	10038	106	**14.8 (12.1 - 17.9)**	670	1	-	2815	13	**4.8 (2.6 - 8.2)**
STS	14453	41	**84.5 (60.6 - 114.6)**	10500	44	**107.8 (78.3 - 144.7)**	675	0	-	2838	4	**32.5 (8.7 - 83.2)**
Bone	14534	40	**312.9 (223.5 - 426.1)**	10522	36	**407.6 (285.5 - 564.4)**	670	1	-	2842	3	**115.5 (23.2 - 337.5)**
CNS	14698	3	**6.4 (1.3 - 18.7)**	10678	3	**8.9 (1.8 - 26.1)**	675	0	-	2851	0	-
Female Breast	7021	2	-	5209	7	**4.5 (1.8 - 9.3)**	337	0	-	1262	3	**6.8 (1.4 - 19.9)**
Oral cavity[d]	14708	0	-	10606	5	**21.6 (7 - 50.5)**	675	0	-	2851	0	-
Nasal cavity[e]	14678	9	**489.1 (223.2 - 928.6)**	10671	2	-	675	0	-	2851	0	-
Uterine corpus	7010	1	-	5273	0	-	337	0	-	1264	1	-
Kidney	14708	0	-	10654	2	-	675	0	-	2851	0	-
Gastrointestinal	14709	1	-	10678	0	-	675	0	-	2851	0	-
Liver/gallbladder	14708	0	-	10678	0	-	675	0	-	2851	0	-
Lung	14708	2	-	10678	0	-	675	0	-	2850	1	-
Cervix	7035	0	-	5273	0	-	337	0	-	1266	0	-
Ovary	7035	0	-	5273	0	-	337	0	-	1266	0	-
Thyroid	14654	3	4.9 (1 - 14.4)	10678	0	-	675	0	-	2851	0	-
Bladder	14697	1	-	10678	0	-	675	0	-	2851	0	-
Melanoma	14580	15	19 (10.6 - 31.4)	10499	12	**19.2 (9.9 - 33.6)**	675	0	-	2849	1	-
Pancreas	14710	1	-	10678	0	-	675	0	-	2851	0	-
Prostate	7673	0	-	5405	0	-	338	0	-	1585	0	-
Hematologic	14701	1	-	10662	3	2.5 (0.5 - 7.3)	675	0	-	2836	2	-
Pineoblastoma	14706	8	**3530 (1520 - 6956)**	10678	0	-	675	0	-	2851	0	-
Other/unspecified	14708	4	**4.9 (1.3 - 12.6)**	10656	3	**5.4 (1.1-15.7)**	675	0	-	2851	0	-

SMN=subsequent malignant neoplasm; STS=soft tissue sarcoma; CNS=central nervous system; Obs=Observed number of SMNs; PY=person-years; SIR=standardized incidence ratio; CI=confidence interval; CT=chemotherapy; RT=radiotherapy; uk=unknown

a. Patients were followed from retinoblastoma diagnosis until earliest of first incident subsequent malignant neoplasm of interest or date of last contact (defined as the most recent questionnaire or, for patients who never completed a questionnaire, the earliest of death or estimated medical record abstraction date).

b. SIR = standardized incidence ratio; observed/expected where the expected numbers are derived from SEER 9 rates 1975-2016 (with rates from 1975-1979 applied for earlier years), stratified by calendar year (1975-1979, 1980-1984, … 2010-2016), sex, and age (0-4, 5-9,…80-84, 85+), and multiplied by stratum-specific person-years at risk in the cohort. SIRs are not shown when observed <3. Bolded values indicate statistically significant SIRs (corresponding to exclusion of 1.0 from the confidence limit).
c. SMN total includes all subsequent malignant neoplasms excluding retinoblastoma, orbit and non-melanoma skin cancer.
d. Includes oral cavity and pharynx
e. Includes nasal cavity, middle ear and sinus.

Supplementary Table 8. Risk factors for developing a subsequent malignant neoplasm among 924 nonhereditary retinoblastoma survivors

Factors	SMN total[a]	
	N	HR (95% CI)[b]
Total	*25*	
Age at retinoblastoma diagnosis		
<12 months	*8*	1.0 (REF)
12-23 months	*8*	0.7 (0.3 – 2.0)
24+ months	*9*	0.4 (0.2 - 1.2)
P-trend[c]		*0.09*
Year of retinoblastoma diagnosis		
<1960	*18*	1.0 (REF)
1960-1969	*6*	1.0 (0.4 - 2.5)
1970-2006	*1*	0.2 (0.02 - 1.9)
P-trend[c]		*0.25*
Sex		
Male	*8*	1.0 (REF)
Female	*17*	1.7 (0.7 - 3.9)
P-value[d]		*0.23*
Family history of retinoblastoma		
No/unknown	*25*	
Yes	*0*	
P-value[d]		
Treatment for retinoblastoma		
RT, no/uk CT	*2*	1.0 (REF)
RT and CT	*1*	1.1 (0.1 - 12.4)
CT, no/uk RT	*1*	1.4 (0.1 - 17.4)
No/uk RT or CT	*21*	0.7 (0.2 – 3.0)
P-value[d]		*0.84*

SMN=subsequent malignant neoplasm; HR=hazard ratio; CI=confidence interval; CT=chemotherapy; RT=radiotherapy; uk=unknown;

a. SMN total includes all subsequent malignant neoplasms excluding retinoblastoma, orbit and non-melanoma skin cancer.
b. Hazard ratios (HRs) and 95% confidence intervals (CI) were estimated from Cox proportional hazards models, mutually adjusted for factors in the table. Patients were followed from retinoblastoma diagnosis until earliest of first incident subsequent malignant neoplasm of interest or date of last contact (defined as the most recent questionnaire or, for patients who never completed a questionnaire, the earliest of death or estimated medical record abstraction date).
c. Wald P-value was obtained by treating the ordinal variable as a continuous variable (with all other variables modelled categorically).
d. Wald P-value for the categorical variable in the model (with all variables modelled categorically).

Supplementary Table S9. Risk for subsequent malignant neoplasms among hereditary and nonhereditary retinoblastoma survivors compared with that in the general population, based on combined incident- and mortality-based cases[a].

	Hereditary (N=1128)					Non-hereditary (N= 924)				
SMN Type	Obs	% total	N known histology[b]	SIR (95% CI)[d]	AER[c]	Obs	% total	N known histology[b]	SIR (95% CI)[c]	AER[d]
SMN Total[e]	369 (429)[f]	100%		**15.4 (13.8 - 17)**	117.7	45(49)[f]	100%		1.3 (0.9 - 1.7)	3.3
STS	129	30%	96	**108.1 (90.3 - 128.5)**	42.0	4	8%	2	3.1 (0.8 - 8)	1.0
Bone	109	25%	86	**400.4 (328.8 - 483)**	35.4	0	0%	NA		
CNS	19	4%	10	**18.3 (11 - 28.6)**	5.8	2	4%	1		
Breast	15	3%	12	**3.2 (1.8 - 5.2)**	3.3	13	27%	8	**1.9 (1.0 - 3.2)**	2.2
Female breast	15		12	**3.2 (1.8 - 5.3)**	7.0	12		8	1.8 (0.9 - 3.1)	3.6
Oral cavity[g]	8	2%	5	**10.1 (4.3 - 19.9)**	2.3	0	0%	NA		
Nasal cavity[h]	15	3%	9	**331.9 (185.6 - 547.4)**	4.8	0	0%	NA		
Uterine corpus	8	2%	4	**11.2 (4.8 - 22.1)**	4.9	1	2%	1		
Kidney	4	1%	2	**4.4 (1.2 - 11.4)**	1.0	1	2%	1		
Gastrointestinal	4	1%	2	1.5 (0.4 - 3.8)	0.4	4	8%	3	1 (0.3 - 2.6)	0.0
Liver/gallbladder	1	0%	0	-		0	0%	NA		
Lung	13	3%	4	**6.3 (3.4 - 10.8)**	3.5	5	10%	1	1.5 (0.5 - 3.4)	0.6
Cervix	2	0%	0	-		1	2%	0		
Ovary	2	0%	0	-		0	0%	NA		
Thyroid	4	1%	3	2.7 (0.7 - 6.9)	0.8	3	6%	3	1.8 (0.4 - 5.3)	0.5
Bladder	8	2%	2	**11.4 (4.9 - 22.4)**	2.3	1	2%	1		
Melanoma	33	8%	30	**16.7 (11.5 - 23.4)**	10.1	4	8%	3	1.7 (0.5 - 4.4)	0.6
Pancreas	5	1%	1	**11.8 (3.8 - 27.5)**	1.5	1	2%	0		
Prostate	1	0%				1	2%	1		
Hematologic	6	1%	5	1.6 (0.6 - 3.4)	0.7	2	4%	2		
Pineoblastoma	8	2%	8	**1798 (774.3 - 3544)**	2.6	0	0%	NA		
Other/unspecified	35	8%	5	**18.5 (12.9 - 25.7)**	10.6	6	12%	1	2.8 (1.0 - 6.1)	1.4

SMN=subsequent malignant neoplasm; STS=soft tissue sarcoma; CNS=central nervous system; Obs=Observed number of SMNs; SIR=standardized incidence ratio; AER=absolute excess risk; CI=confidence interval

a. Patients were followed from retinoblastoma diagnosis until earliest of first SMN of interest (based on date of diagnosis or date of death for cancers ascertained from NDI/death certificates), death, or date of last known contact.

b. Number of cases with ICD-O-3 morphology codes excluding 8000-8001.
c. SIR = standardized incidence ratio; observed/expected where the expected numbers are derived from SEER 9 rates 1975-2016 (with rates from 1975-1979 applied for earlier years), stratified by calendar year (1975-1979, 1980-1984, … 2010-2016), sex, and age (0-4, 5-9,…80-84, 85+), and multiplied by stratum-specific person-years at risk in the cohort. SIRs and AERs not shown when observed <3. Bolded values indicate statistically significant SIRs (corresponding to exclusion of 1.0 from the confidence limit).
d. AER = absolute excess risk per 10,000 person-years ([(obs - expected) / person-years] x 10,000).
e. SMN total includes all subsequent malignant neoplasms excluding retinoblastoma, orbit and non-melanoma skin cancer.
f. Number observed represents the number of individuals who developed any SMN (i.e., the first SMN). The number in parentheses represents the total number of SMNs, counting multiple SMNs per person but only the first occurrence of each type of SMN.
g. Includes oral cavity and pharynx.
h. Includes nasal cavity, middle ear and sinus.
